# Optimization of Polycistronic Anti-CCR5 Artificial microRNA Leads to Improved Accuracy of Its Lentiviral Vector Transfer and More Potent Inhibition of HIV-1 in CD4^+^ T-Cells

**DOI:** 10.3390/cells7020010

**Published:** 2018-02-04

**Authors:** Felix Urusov, Dina Glazkova, Denis Omelchenko, Elena Bogoslovskaya, Galina Tsyganova, Katerina Kersting, German Shipulin, Vadim Pokrovsky

**Affiliations:** Department of Molecular Diagnostic and Epidemiology, Federal Budget Institution of Science “Central Research Institute of Epidemiology” of The Federal Service on Customers’ Rights Protection and Human Well-being Surveillance, 111123 Moscow, Russia; dina.glazkova@pcr.ru (D.G.); omdeno@gmail.com (D.O.); lenabo@pcr.ru (E.B.); dialogalaine@mail.ru (G.T.); ketrin08@mail.ru (K.K.); shipgerman@gmail.com (G.S.); v.pokrovskiy@hiv-russia.ru (V.P.)

**Keywords:** HIV gene therapy, CCR5 co-receptor, RNA interference, miRNA, lentiviral vectors

## Abstract

C-C chemokine receptor type 5 (CCR5) is utilized by human immunodeficiency virus (HIV) as a co-receptor for cell entry. Suppression of the CCR5 gene by artificial microRNAs (amiRNAs) could confer cell resistance. In previous work, we created a lentivector that encoded the polycistron of two identical amiRNAs that could effectively suppress CCR5. However, tandem repeats in lentiviral vectors led to deletions of the repeated sequences during reverse transcription of the vector RNA. To solve this problem, we have created a new amiRNA against CCR5, mic1002, which has a different microRNA scaffold and targets a different sequence. Replacing one of the two identical tandem amiRNAs in the polycistron with the mic1002 amiRNA increased the accuracy of its lentiviral vector transfer while retaining its ability to effectively suppress CCR5. A lentiviral vector containing two heterogenic amiRNAs significantly inhibited HIV replication in a vector-transduced human CD4^+^ lymphocyte culture.

## 1. Introduction

The spread of human immunodeficiency virus (HIV) infection is of great concern worldwide. Antiretroviral drugs, which HIV patients must take for their entire lives, can be toxic and do not always prevent disease progression due to the emergence of resistant HIV strains. Therefore, new treatment approaches are being sought, including a gene therapy-based approach involving the genetic modification of cells susceptible to HIV to promote their viral resistance. This could lead to long-term infection control without chemotherapy or even to a complete cure.

A promising strategy for HIV gene therapy is downregulating the expression of CCR5, one of two co-receptors for viral cell entry [1]. The genetic deficiency of CCR5 in individuals bearing mutant alleles confers resistance to HIV infection, while people lacking CCR5 seem perfectly healthy [2,3,4,5].

Knockout of CCR5 can be accomplished by transient expression of nucleases with a programmed specificity in target cells [6]. Also, RNA interference (RNAi) can be used to suppress CCR5 [7]. Short interfering RNAs (siRNA) derived from short hairpin RNAs (shRNAs) precursors can effectively suppress CCR5 [8,9]. A vector that includes shRNA against CCR5 is currently being tested in clinical trial [10]. However, excessive amounts of siRNAs or their precursors could be toxic to cells [11,12]. Because artificial microRNAs (amiRNAs) do not exhibit toxicity [13], they are more suitable for gene therapy. Genes encoding amiRNAs are created by replacing the native antisense strands of endogenous miRNA genes with sequences targeted to genes of interest.

Several amiRNA genes can be clustered and transcribed as single polycistronic transcripts. So-called polycistronic amiRNAs are reminiscent of clusters of miRNA found in the human genome and induce more potent gene silencing [14,15]. To date, a triple amiRNA polycistron has been designed against CCR5, and NSG mice transplanted with the cells bearing this combination have been shown to be resistant to a CCR5-tropic HIV infection [16].

We previously developed several lentiviral constructs encoding anti-CCR5 amiRNAs. Each construct was tested for its capacity to silence CCR5. One of them, the mic13lg + mic13lg construct, which contained two identical amiRNAs, showed the best results [17]. However, tandem repeat sequences in lentiviral constructs can potentially lead to deletions in the vector [18,19]. Lentiviruses contain two genomic RNA molecules, and reverse transcriptase can switch between two matrices during conversion RNA genome into DNA [18,19]. This could lead to the skipping of some repeating sequences. Additionally, the presense of amiRNA hairpins in lentiviral vectors can increase the likelihood of rearrangement, because reverse transcriptase presumably pauses in RNA regions with stable secondary structures and thus promotes the template switching [18]. Deletions led to reduced vector inhibitory properties, as it was shown for the lentivector, which contained multiple anti-HIV shRNA [20]. Thus, the presence of tandem repeats in the lentivector is undesirable. 

In this study, we optimized the mic13lg + mic13lg tandem amiRNA by replacing one of the two identical mic13lg sequences with another amiRNA against CCR5 (mic1002), which recognizes another region of the CCR5 gene. The main goal of this study was to compare vectors with the tandem identical amiRNAs and the different amiRNAs in terms of the genetic stability of the vector during the transduction process and anti-HIV activity. 

## 2. Materials and Methods 

### 2.1. Cells and Plasmid DNA 

HEK 293FT (Invitrogen, Thermo Fisher Scientific, Waltham, MA, USA) and MAGI CCR5 cells were cultured in Dulbecco’s Modified Eagles Medium supplemented with 4 mM L-GlutaMAX-I (Gibco, Thermo Fisher Scientific, Waltham, MA, USA) and 10% fetal bovine serum (FBS, Clontech, Mountain View, CA, USA). The MAGI CCR5 cells were obtained from the NIH AIDS Research and Reference Reagent Program (National Institutes of Health, Bethesda, MD, USA). Peripheral blood mononuclear cells (PBMC) were isolated using the Lympholyte-Mammal reagent (Cedarlane, Burlington, ON, Canada), and CD4^+^ lymphocytes from PBMCs were isolated using the EasySep Human CD4^+^ T cell enrichment kit (STEMCELL Technologies, Vancouver, BC, Canada). Primary CD4^+^ lymphocytes were activated by CD3/CD28 Dynabeads Human T-Activator (Gibco, Thermo Fisher Scientific, Waltham, MA, USA) and cultured in X-VIVO15 medium (Lonza, Basel, Switzerland) containing 100 IU/mL interleukin-2 (Biotech, St. Petersburg, Russia). HT1080 CCR5-EGFP cells [17] were cultured in Minimum Essential Medium supplemented with 10% FBS. Then, all were cultured at 37 °C and 5% CO_2_. Construction of vectors plasmids mic1001-Puro, mic1001lg-Puro, mic1002-Puro, mic1002 + mic13lg-Puro, mic13lg + mic1002-Puro, mic13lg + mic13lg-Puro, mic1002-EGFP, mic13lg + mic13lg-EGFP, mic1002 + mic13lg-EGFP, and mic13lg + mic1002-EGFP is described in Supplementary Methods.

### 2.2. Lentiviral Particle Production

Pseudovirus particle preparation and infectious titer determination were performed according to a previously described protocol [17]. Briefly, HEK 293FT cells were cotransfected with the lentiviral vector plasmids and Lenti-X HT Packaging mix (Clontech, Mountain View, CA, USA). Culture media containing pseudoviral particles was collected 60 h after transfection, and the initial titer for all the lentiviral vectors was approximately 5 × 10^6^ TU/mL. For transduction of CD4^+^ primary lymphocytes, lentiviral particles were concentrated by ultracentrifugation on a 20% sucrose cushion for 2 h at 25,000 rpm at 4 °C with an Optima L-90K ultracentrifuge (Beckman Coulter, Brea, CA, USA) and an SW-32Ti rotor. 

### 2.3. Lentiviral Transduction 

HT1080 CCR5-EGFP and MAGI CCR5 cells were transduced in the presence of 4 μg/mL or 8 μg/mL polybrene, respectively. Selection of transduced HT1080 CCR5-EGFP cells was achieved with 1 µg/mL puromycin. CD4^+^ cells were transduced 24 h after CD3/CD28 activation in the presence of 6 μg/mL polybrene. 

### 2.4. Flow Cytometry 

Flow cytometry experiments were performed on a FACS Canto II flow cytometer (BD, Franklin Lakes, NJ, USA), and data were analyzed using Flowing Software 2.5.1 (Centre for Biotechnology University of Turku, Finland, http://www.uskonaskel.fi/flowingsoftware/). An NP-6G4 monoclonal antibody labeled with PerCP-eFluor 710 (eBioscience Thermo Fisher Scientific, Waltham, MA USA) was used to measure CCR5 on the cell surface. 

### 2.5. PCR Analysis of Vector Integrity 

To assess vector integrity, we amplified the vector sequence from the НТ1080-CCR5-EGFP genome using the CMV end for primer (5′-GGTAGGCGTGTACGGTGGGAG-3′) and the WPRE rev primer (5′-ATTGAGGGCCGAAGGGACGTAGC-3′). Nested PCR was used to analyze CD4^+^ cells using the cPPT for (5′-AGTACAAATGGCAGTATTCATCC-3′) and WPRE rev (5′-ATTGAGGGCCGAAGGGACGTAGC-3′) outer primers, and the Ef1a end for (5′-CATTCTCAAGCCTCAGACAGTGGTTC-3′) and Ppgk rev (5′-AATGTGTGCGAGGCCAGAGG-3′) nested primers. 

### 2.6. Quantitative PCR (qPCR)

qPCR was performed using a Rotor-Gene 3000 amplifier (Corbett Research, Sydney, Australia), and DNA/RNA isolation was achieved using the AmpliSens RIBO-prep kit (AmpliSens, Moscow, Russia). *CCR5* mRNA was quantified by qPCR using the β-glucuronidase gene as a reference. For qPCR analysis of mature miRNA, total RNA (including small RNAs) was isolated using the miRNeasy Mini Kit (Qiagen, Hilden, Germany). The mic13lg amiRNA was quantified as described in Chen et al. [21] using the following primers: mic13lg reverse transcription primer (5′-GTCGTATCCAGTGCAGGGTCCGAGGTATTCGCACTGGATACGA-CGTGTCA-3′), mic13lg forw (5′-CCAGGAATTGATGTCATAG-3′), and mic13lg rev (5′-GTGCAGGGTCCGAGGT-3′). The QuantiMir RT kit (System Biosciences, Palo Alto, CA, USA) and the mic1002 forward primer (5′-ATCGGGTGTAAACTGAGCT-3′) were used to measure mic1002. For both amiRNAs, U6 small nuclear RNA was used as a reference. 

### 2.7. HIV Challenge

NL(AD8), an HIV-1 AD8 Macrophage-tropic R5 virus obtained from the NIH AIDS Research and Reference Reagent Program in the USA, was used to infect cells. MAGI CCR5 cells were splited 1:20 24 h before HIV challenge. Before infection, the cell culture media was removed and 1 mL of fresh media containing 1 ng of virus was added to the cells in a 25 cm^2^ flask. The infected cells were incubated at 37 °C for 2 h with stirring every 30 min. The cells were washed twice with serum-free medium, and 5 mL of fresh culture medium was added. Every two days 2 mL of medium was replaced with fresh media. The removed medium was used for HIV RNA analysis. The HIV challenge of CD4^+^ lymphocytes was conducted 5 days after activation. Briefly, 10^6^ CD4^+^ lymphocytes were mixed with 10 μL of viral suspension containing 100 ng/mL p24 antigen in 1 mL of media. The infected cells were incubated at 37 °C for 2 h with stirring every 30 min. The cells were washed with serum-free medium twice and resuspended in complete culture medium. The lymphocytes were counted and diluted with fresh medium at a density of 0.7 × 10^6^ cells per ml twice a week. The concentrations of HIV RNA were determined in the culture supernatant using the AmpliSens HIV Monitor-FRT reagent kit (AmpliSens, Mоsсow, Russia).

## 3. Results 

### 3.1. Design of the New amiRNAs and Evaluation of Their Silencing Activity 

**Figure 1:**
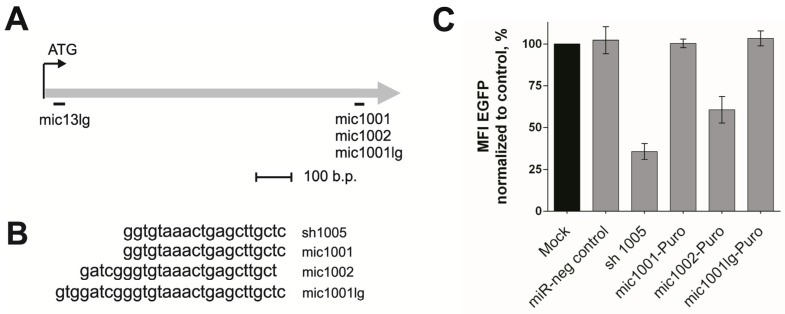
Creation of the anti-CCR5 amiRNAs on the mir20 backbone and testing of their knockdown efficiency. (**A**) Open reading frame of the CCR5 gene. The mic13lg, mic1001, mic1002, and mic1001lg amiRNA recognition sites are depicted; (**B**) antisense chain sequences of mic1001, mic1002, and mic1001lg; (**С**) estimation of the mic1001, mic1002, and mic1001lg efficiency on HT1080 ССR5-EGFP cells. Sh1005 was used as a positive control. miR-neg control vector was used as a nonsilensing negative control. The error bars show ± SEM (*n* = 3).

The endogenous human miRNA mir20 (part of the mir17–92 polycistron) was chosen as the scaffold for creating new amiRNAs because of its previously demonstrated good functionality as an amiRNA in polycistrons [15]. To design new amiRNAs, the original mir20 antisense strand was replaced with the anti-CCR5 target sequence, the CCR5 coding sequence (CDS) region located 1001–1002 bp downstream from the CDS start site. Targeting this site with sh1005 efficiently suppressed CCR5 expression and exhibited low toxicity [9]. Three new amiRNAs were created targeting these regions of CCR5, mic1001, mic1002, and mic1001lg (Figure 1a), which differed in their lengths of antisense strands by 20, 24, and 28 bp, respectively (Figure 1b), as this length can significantly influence amiRNA efficiency [8,17]. These amiRNAs were included in the LTR VECT lentiviral vector [22] with the puromycin resistance marker.

To test the silencing activity of the new amiRNAs, we used HT1080 CCR5-EGFP indicator cells with an imbedded transgene encoding the CCR5-EGFP chimeric protein [17]. Downregulation of CCR5-EGFP expression by amiRNAs against CCR5 can be easily quantified by detecting decreased EGFP fluorescence. We transduced HT1080 CCR5-EGFP cells with the mic1001-Puro, mic1001lg-Puro, and mic1002-Puro lentiviral vectors encoding amiRNAs. A low multiplicity of infection (MOI, 0.1) was used to ensure all the transduced cells carried only one copy of the vector after puromycin selection, which was verified by qPCR (Figure S1). Suppression of the CCR5-EGFP transgene by an equal vector copy number allowed the lentiviral constructs to be correctly compared. The amiRNAs mic1001 and mic1001lg (20 and 28 nt, respectively) did not suppress CCR5 expression, while mic1002 (24 nt) reduced CCR5-EGFP expression on the cell surface by 40% (Figure 1c). Thus, among three new amiRNAs only mic1002 exhibited silencing activity, and this amiRNA was selected for further development of hybrid constructs.

### 3.2. Creation of the Tandem Constructs and Evaluation of Their Silencing Activity and Integrity

**Figure 2:**
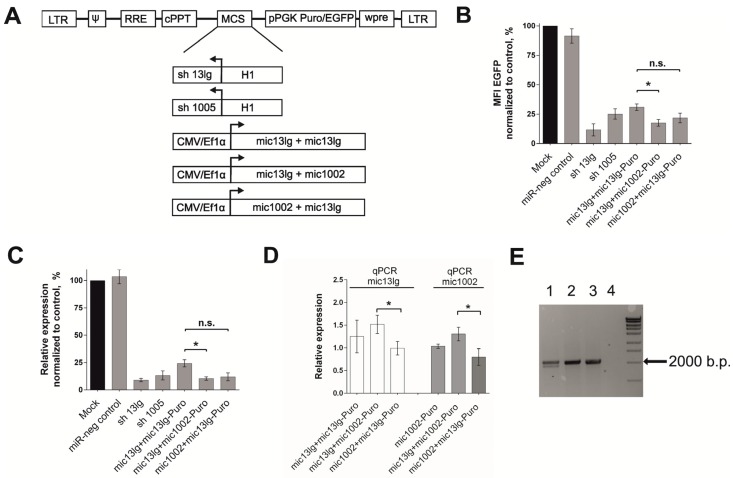
Evaluation of the effectiveness and integrity of the amiRNA vectors on НТ1080-CCR5-EGFP cells. (**A**) Diagram of the lentiviral constructs used in this work. LTR—long terminal repeat, Ψ—lentiviral package signal, RRE—Rev responsive element, cPPT—central polypurine track, pPGK phosphoglycerate kinase promoter, WPRE—woodchuck posttranscriptional regulatory element, and MCS—multiple cloning site. Н1—shRNA promoter (pol III promoter). To express the amiRNAs, CMV or Ef1б promoters were used. The puromycin resistance gene (*Puro*) or green fluorescent protein gene (*EGFP*) were used as markers; (**B**) ratio of the mean fluorescent intensity (MFI) of amiRNAs transduced cells to that of the mock transduced cells (*n* = 5); (**C**) quantitative analysis of CCR5 mRNA in НТ1080-CCR5-EGFP cells. The CCR5 mRNA level in amiRNAs transduced cells was normalized to that of control mock transduced cells (*n* = 5); (**D**) quantitative analysis of mature amiRNAs in transduced НТ1080-CCR5-EGFP cells. Mature amiRNA levels were normalized to that of the U6 small nuclear RNA (*n* = 3); (**E**) integrity analysis of the vectors inserted into the НТ1080-CCR5-EGFP cellular genome. Lane 1—mic13lg+mic13lg-Puro, lane 2—mic13lg+mic1002-Puro, lane 3—mic1002+mic13lg-Puro, lane 4—negative PCR control. All the error bars show ± SEM. n.s.—not significant (*p* > 0.05 using the Mann-Whitney test), * *p* < 0.05 using the Mann-Whitney test.

We created two constructs that contained two different amiRNAs, mic13lg, and mic1002. A brief description of the initial amiRNAs is presented in Table S2. The new combinations in the mic13lg + mic1002-Puro and mic1002 + mic13lg-Puro lentiviral vectors differed in their amiRNA arrangements, and diagrams of all the lentiviral vectors used in the work are shown in Figure 2a.

The silencing activities of the mic13lg + mic1002-Puro and mic1002 + mic13lg-Puro constructs were assessed in HT1080 CCR5-EGFP cells as described above. Vectors carrying sh13lg and sh1005 were used in the experiments as controls. As seen in Figure 2b, both newly created constructs effectively suppressed CCR5-*EGFP* transgene expression. The silencing activity of mic13lg + mic1002 was higher than that of the previously described mic13lg + mic13lg.

Additionally, we quantified CCR5-EGFP mRNA expression (Figure 2c) in vector-transduced HT1080 CCR5-EGFP cells. The level decreased in a manner similar to that of EGFP fluorescence (Figure 2b), showing that regulation of the CCR5-*EGFP* transgene occurred at the RNA level. 

Because the rate of effector amiRNA production directly affects the knockdown activity of amiRNA polycistrons, we quantified mature amiRNAs in mic13lg + mic1002-Puro- and mic1002 + mic13lg-Puro-transduced cells using qPCR. The mic13lg levels in cells transduced with both new constructs were comparable to those in cells transduced with the mic13lg + mic13lg-Puro double construct (Figure 2d) despite the fact that only one copy of mic13lg existed in the mic13lg + mic1002 and mic1002 + mic13lg tandem amiRNAs. When comparing the new constructs to each other, mic13lg and mic1002 expression was higher in the mic13lg + mic1002-Puro construct (Figure 2d). More efficient mic13lg production in the new constructs may be explained by the presence of mic1002 in the polycistron, which can provide an optimal secondary structure for processing the amiRNA precursor (Figure S2). Enhancing the production of some miRNAs by using them in combination with other miRNAs in polycistrons has been previously described [15].

The decreased levels of mature microRNA13lg may also be due to the instability of the mic13lg + mic13lg tandem amiRNA and loss of one of the amiRNAs. To test this hypothesis, we determined the structures of our constructs after they integrated into the host cell genome amplifying the vector regions, which included the amiRNA sequences. Genomic DNA extracted from the transduced cells was used as a matrix. The expected fragment of approximately 2000 bp was detected in all the samples (Figure 2e), and an additional smaller fragment was discovered only in the sample obtained from cells transduced with the mic13lg + mic13lg-Puro construct. The length of this fragment corresponded to the vector in which mic13lg was deleted. No additional PCR products were observed in the samples obtained from cells transduced with the mic13lg + mic1002-Puro and mic1002 + mic13lg-Puro vectors, indicating that unlike the mic13lg + mic13lg-Puro construct, the vectors containing the new amiRNAs combinations are not prone to losses of amiRNAs sequences during processing of lentivector RNA in target cells.

### 3.3. amiRNAs Suppress HIV Growth in MAGI CCR5 and Primary T Cell Cultures

Next, we evaluated the impact of CCR5 amiRNAs on the susceptibility of cells to HIV infection. We used MAGI CCR5 cells for the HIV challenge experiments because they express CCR5 on their surface and are sensitive to R5-tropic HIV infection. Cells were transduced at MOI 1 with following lentiviral vectors: mic13lg + mic1002-EGFP, mic1002 + mic13lg-EGFP, and mic13lg + mic13lg-EGFP, and the percentages of modified cells in cultures were 68.9, 78.6, and 64.9, respectively. 

**Figure 3:**
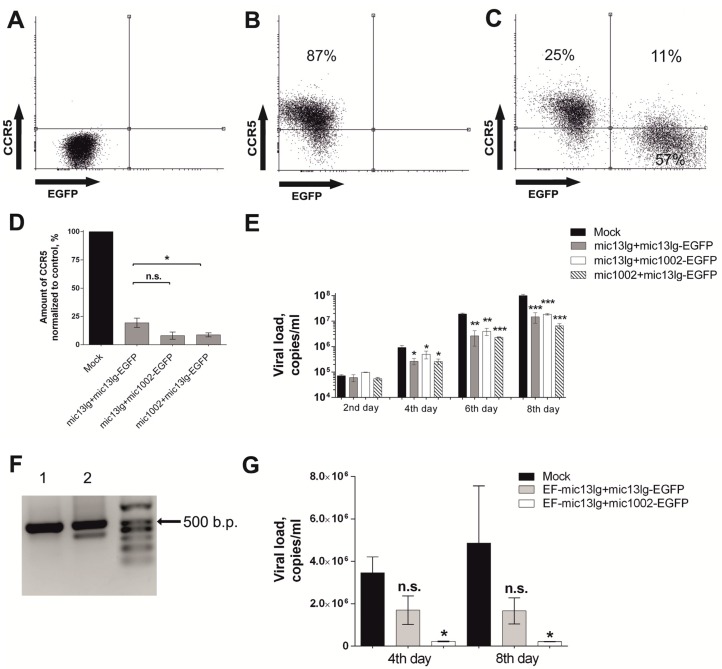
Evaluation of the effectiveness of the amiRNA vectors on MAGI CCR5 and CD4^+^ lymphocytes. (**A**–**D**)—Amount of surface CCR5 on MAGI CCR5 cells transduced with amiRNAs. Flow cytometry data. (**A**)—Untransduced cells without antibody staining; (**B**)—untransduced cells stained with antibodies against ССR5; (**C**)—cells transduced with mic13lg + mic1002 and stained with antibodies against ССR5; (**D**)—amount of CCR5 on the surface of amiRNA-modified cells (EGFP+, left top, and bottom quadrants in Figure 3с) calculated as the % modified cells Ч MFI of antibodies labeled with PerCP-eFluor 710; the error bars show ± SEM (*n* = 3), n.s.—not significant (*p* > 0.05 using the Mann-Whitney test), * *p* < 0.05 using the Mann-Whitney test; (**E**)—viral load in infected MAGI CCR5 cell cultures compared with the mock control, * *p* <0.05, ** *p* <0.001, *** *p* < 0001 according *t*-test. The error bars show ± SEM (*n* = 3); (**F**)—integrity analysis of the vectors inserted into the CD4^+^ cellular genome. Lane 1—EF-mic13lg + mic1002-EGFP, lane 2—EF-mic13lg + mic13lg-EGFP; (**G**)—challenge experiments with R5-tropic HIV-1. The viral loads in cultures of infected CD4+ lymphocytes are indicated. The error bars show ± SEM (*n* = 3), n.s.—not significant (*p* > 0.05 using the Mann-Whitney test), * *p* < 0.05 using the Mann-Whitney test.

To assess the influence of the various amiRNAs on the level of surface CCR5, transduced cells were stained with fluorescently labeled antibodies against CCR5. The percentage of cells containing CCR5 and the mean fluorescence intensity (MFI) of the CCR5-positive cells were measured by flow cytometry (Figure 3a–d). CCR5 expression was significantly decreased in cells containing any of the tandem amiRNA combinations (Figure 3d). Furthermore, the new constructs were approximately 2 times more efficient than mic13lg + mic13lg-EGFP (Figure 3d), but statistically significant differences were shown only for mic1002 + mic13lg-EGFP.

The modified cell cultures were infected with the AD8 strain of R5-tropic HIV, and during the experiment, HIV RNA concentrations were measured in the infected cultures. All of the constructs could suppress viral growth to a roughly equal extent (Figure 3e).

Next, we tested the effectiveness of our constructs on primary human CD4^+^ lymphocytes, which are potential targets for HIV gene therapy. For these experiments, the CMV promoter in the vectors was replaced with an EF1α promoter, which provides more stable expression in human cells [23]. Because CD4^+^ lymphocytes are difficult to transduce, we used a higher MOI of 10 to achieve efficient transduction. Using these conditions, transduction of CD4^+^ cells with the EF-mic13lg + mic1002-EGFP and EF-mic13lg + mic13lg-EGFP lentiviral constructs resulted in cultures with 39 and 38% of the cells being modified, respectively. Like in HT1080 CCR5-EGFP cells, deletions in the integrated EF-mic13lg + mic13lg-EGFP vectors sequences were observed in the CD4^+^ lymphocytes, as an additional smaller PCR fragment was detected (Figure 3f). Sequence analysis confirmed the deletion of one amiRNA in short fragments (Figure S3). The modified cells were infected with R5-tropic HIV, and the viral load in the EF-mic13lg + mic1002-EGFP-transduced cells was significantly lower than that in the unmodified control cells (Figure 3g). The difference in the viral load between the EF-mic13lg + mic13lg-EGFP-transduced cells and the control cells was not significant. Together, these results indicate that the EF-mic13lg + mic1002-EGFP vector can protect human CD4^+^ cells from HIV. 

## 4. Discussion 

Lentiviral delivery of the genetic factors protecting cells from HIV is one of the promising strategies for HIV gene therapy. An approach which is aimed at several targets and allows for the prevention of HIV-provirus integration is believed to be the most effective. CCR5 receptor is a well-studied target, and its removal from the cell surface prevents R5-tropic HIV from entering the cells. Downregulation of CCR5 can be achieved by means of RNA interference, namely by amiRNA expression, which is a non-toxic and efficient tool for gene silencing [13]. In the previous work, we described the tandem of two identical amiRNAs that could effectively inhibit CCR5 [17]. However, the presence of the repeating sequence in the lentiviral vector raised doubts about the accuracy of gene transfer [18,19,20].

Herein, we have shown that two identical amiRNAs in the previously described lentiviral vector caused deletions in the integrated copy of the vector. We optimized the polycistronic sequence to improve its stability during the lentiviral delivery. As a result, two new amiRNA polycistrons, mic13lg + mic1002 and mic1002 + mic13lg, which do not contain tandem repeat sequences, were designed. Implementing optimization was shown to prevent deletions of vector elements (amiRNA) in indicator cells, as well as in CD4^+^ human lymphocytes.

The amiRNAs of the new polycistrons target two different regions of the CCR5 gene, which can help to preserve the inhibitory activity of the vector if one of the two amiRNAs reduces functionality due to a CCR5 gene polymorphism. It is known that even a single nucleotide substitution can affect the RNAi process [24]. In RNAi studies there is a trend towards increasing the number of targets as it was done for anti-HIV short RNAs [15,20,25]. Notably, many single nucleotide polymorphisms have been described for the CCR5 gene [26,27], but in most of anti-CCR5 RNAi studies a single target is used [7,8,9,16]. Therefore, increasing the number of anti-CCR5 amiRNAs targets is reasonable. 

The silencing activity of the constructs was tested in various experiments and was estimated by the suppression of the CCR5-EGFP transgene in HT1080 CCR5-EGFP cells (EGFP fluorescence), reduced CCR5 RNA levels in the same cells, and direct quantification of the CCR5 receptor on the surface of MAGI CCR5 cells. The effectiveness of the new polycistrons was comparable to or higher than that of mic13lg + mic13lg. The effectiveness of the new constructs was sufficient to protect cells from HIV in vitro, as demonstrated by the virological experiments with the R5-tropic virus in MAGI CCR5 cells and in primary human CD4^+^ lymphocytes. The degree of HIV inhibition in mic13lg + mic13lg-transduced MAGI CCR5 cells and in MAGI CCR5 cells transduced with new polycistrons was similar. However, there was a significant difference between antiviral activity of EF-mic13lg + mic13lg-EGFP and EF-mic13lg + mic1002-EGFP constructions in primary CD4^+^ lymphocytes, which emphasizes the importance of antiviral construct activity testing in primary human cells. So, in the study of Myburgh et al., the efficient HIV inhibition by triple anti-CCR5 amiRNA was demonstrated in the cell culture originated from HeLa cell line [28], but in experiments with mice humanized with hCD34^+^ cells no differences in viral load were observed between mice transplanted with modified cells and the control group [16]. These results can be explained by the deletion of amiRNA sequence during the lentiviral transfer of genetic material. One or two amiRNA copies may have been deleted as tandem repeating sequences, which reduced the efficiency of the vector. The vector integrity after insertion in the target cells was not checked in this work. Only when the mice had been transplanted with sorted CD34^+^ cells containing 90% of transduced cells the protection of animals from HIV infection was demonstrated.

Importantly, no toxic effects of the generated amiRNA constructs were observed. We had been monitoring the percentage of modified cells in the culture for nearly 1 month and did not observe the decrease in EGFP^+^ cell percentages (Figure S4). 

Thus, the newly created anti-CCR5 microRNA polycistrons are suitable for further development as a part of a complex drug for HIV gene therapy. It should be noted that knockdown of CCR5 can protect cells only from R5-tropic virus, but not from X4- or dual-tropic viruses. Therefore, the combination of amiRNA polycistron with additional anti-viral genes in one lentiviral vector will be required to protect cells from all variants of the virus. Besides, this work demonstrated the potential of amiRNA tool, the advantage of using miRNA polycistron for gene silencing, and the importance of vector structure for the lentiviral transfer. 

## Supplementary Materials

The following are available online at http://www.mdpi.com/2073-4409/7/2/10/s1. Supplementary Methods describe plasmid construction; Table S1: Oligonucleotides sequences for plasmid cloning; Table S2: characteristics of mic13lg and mic1002 amiRNAs; Figure S1: average transgene copy number per cell data; Figure S2: percentage of EF-mic13lg + mic1002-EGFP vector-modified CD4^+^cells (EGFP^+^) during one month of the cells cultivation.

## Abbreviations

HIV	human immunodeficiency virus
amiRNA	artificial microRNA
miRNA	microRNA
shRNA	short hairpin RNA
RNAi	RNA interference
siRNA	short interfering RNA
