# Supplementary material for: Optimization of Polycistronic Anti-CCR5 Artificial microRNA Leads to Improved Accuracy of Its Lentiviral Vector Transfer and More Potent Inhibition of HIV-1 in CD4+ T-Cells
Source: Cells. 2018 Feb 4;7(2):10. doi: 10.3390/cells7020010 (PMC5850098; doi:10.3390/cells7020010)
Supplement: Supplementary file 1 [file cells-07-00010-s001.pdf]

**Optimization of polycistronic antiCCR5 artificial microRNA leads to improved accuracy of its lentiviral vector transfer and more potent inhibition of HIV-1 in CD4$^+$ T-cells**

**Felix Urusov, Dina Glazkova, Denis Omelchenko, Elena Bogoslovskaya, Galina Tsyganova, Katerina Kersting, German Shipulin, Vadim Pokrovsky**

Supplementary text and figures:

Supplementary Methods
Supplementary Table S1
Supplementary Table S2
Supplementary Figure S1
Supplementary Figure S2
Supplementary Figure S3
Supplementary Figure S4

**Supplementary Methods**

Plasmid construction. The mic20 flank plasmid, which contained miR20 flanking sequences, was obtained by inserting the miR20 Fla2 rev, miR20 Fla3 for, miR20 Fla1 for, and miR20 Fla4 rev annealed oligos (Table S1) into the pcDNA6.2-GW/miR-neg control plasmid linearized by BamHI and XhoI (Invitrogen). To obtain the miR1001, miR1002, and miR1001lg amiRNA coding sequences, four oligos for each amiRNA (Table S1) were annealed and ligated with the BsmBI-digested mic20 flank plasmid. The BglII/SalI fragment from miR13lg [17] encoding the amiRNA was ligated into the BamHI/SalI sites of miR1002 to generate the tandem miR1002+miR13lg construct, while the BglII/SalI fragment of miR1002 was ligated into the BamHI/SalI of miR13lg to generate the miR13lg+miR1002 tandem construct. Generation of miR13lg+miR13lg and the H1sh13lg rev plasmid with sh13lg was previously described [17]. Four oligos (Table S1) were annealed and inserted into the BglII/HindIII sites of the pRNA-H1/Neo plasmid (Genscreen) to generate the pH1-sh1005 plasmid. Single microRNAs, sh1005 or tandem amiRNAs were transferred to the LTR VECT lentivector [22], the EGFP lentivector (LTR VECT wherein the puromycin resistance gene was replaced with the EGFP reporter), or the EF1α-EGFP lentivector (wherein in addition to the EGFP reporter being replaced, the CMV promoter was replaced by the EF1α promoter).To transfer the constructs into lentiviral vectors, the amiRNA plasmids were digested with BglII/SalI, and the fragments that contained amiRNAs

were cloned into the corresponding BamHI/XhoI sites of the different lentivectors. The resulting vectors were designated mic1001-Puro, mic1001lg-Puro, mic1002-Puro, mic1002+mic13lg-Puro, mic13lg+mic1002-Puro, and mic13lg+mic13lg-Puro for vectors with the CMV promoter and the puromycin marker; mic1002-EGFP, mic13lg+mic13lg-EGFP, mic1002+mic13lg-EGFP and mic13lg+mic1002-EGFP for vectors with the CMV promoter and the EGFP marker; and EF-mic13lg+mic13lg-EGFP and EF-mic13lg+mic1002-EGFP for vectors with the EF1α promoter and the EGFP marker. To transfer sh1005 and sh13lg with the H1 promoter into LTR VECT, the XbaI(blunted)/BglII fragments from plasmids pH1-sh1005 and pH1-CCR5 shRNA [17] were cloned into the BamHI/ClaI (blunted) sites of LTR VECT, generating sh1005 and sh13lg vectors containing the corresponding short hairpins in reverse orientation. The empty vectors LTR VECT, EGFP and EF1α-EGFP were used as corresponding controls As a nonsilensing negative micro RNA control we used lentivector contained miR-neg micro RNA, which is predicted not to target any known vertebrate gene. The sequence of this amiRNA was derived from pcDNA™6.2-GW/miR-neg control plasmid (Invitrogen).

**Supplementary Table S1.** Oligonucleotides sequences

| № | Name | 5’ - 3’ sequence | |
|---|---|---|---|
| 1 | miR20 Fla2 rev | Tagcacgtctctcagaagctgtcacatcagatagaccaggcagattctacatg | For cloning mic20 flank plasmid |
| 2 | miR20 Fla3 for | Gctacgatccgtctcggctagctgtagaactccagcttagatctggccgcac | |
| 3 | miR20 Fla1 for | Gatccatgtagaatctgcctggtctatctgatgtgacagcttctgagagacgt | |
| 4 | miR20 Fla4 rev | Tcgagtgcggccagatctaagctggagttctacagctagccgagacggatcg | |
| 5 | sh1005 O1 for | Gatctccgagcaagctcagtttacaccttgtc | For cloning sh1005 |
| 6 | sh1005 O2 rev | Gtcggacaaggtgtaaactgagcttgctcgga | |
| 7 | sh1005 O3 for | Cgacggtgtaaactgagcttgctcttttt | |
| 8 | sh1005 O4 rev | Tcgaaaaaaagagcaagctcagtttacacc | |
| 9 | Mi1002 -20 O1 for | Tctgtagcagatcgggtgtaaactgagcttgcttgttta | For cloning miR1002 |
| 10 | Mi1002-20 O4 rev | Tagcagtagaaacgggtgcaaactgagctgcta | |
| 11 | Mi1002-20 O3 for | Gtcatagcagctcagtttgcacccgtttctact | |
| 12 | Mi1002-20 O2 rev | Tgactaaacaagcaagctcagtttacacccgatctgcta | |
| 13 | Mi1001 -20 O1 for | Tctgtagcacggtgtaaactgagcttgctcgtagtgtt | For cloning miR1001 |
| 14 | Mi1001-20 O4 rev | Tagcagtacggaagtaaactgaacttgctctagata | |
| 15 | Mi1001-20 O3 for | Tagttatctagagcaagttcagtttacttccgtact | |
| 16 | mi1001-20 O2 rev | Actaaacactacgagcaagctcagtttacaccgtgcta | |
| 17 | Mi1001lg-20 O1 for | Tctgtagcacgtggatcgggtgtaaactgagcttgctcgtagtgtt | For cloning miR1001lg |
| 18 | Mi1001lg-20 O4 rev | Tagcagtacgaaggatcaggtgcaaactgaacttgctctagata | |
| 19 | Mi1001lg-20 O3 for | Tagttatctagagcaagttcagtttgcacctgatccttcgtact | |
| 20 | Mi1001lg-20 O2 rev | Actaaacactacgagcaagctcagtttacacccgatccacgtgcta | |

**Supplementary Table S2.** Characteristics of mic13lg and mic1002 amiRNAs

| Name | AntiCCR5 sequence | miRNA flanking sequence |
|---|---|---|
| mic13lg (28 nt) | aattgatgtcatagattggacttgacac | miR-155 |
| mic1002 (24 nt) | gatcgggtgtaaactgagcttgct | miR-20 |

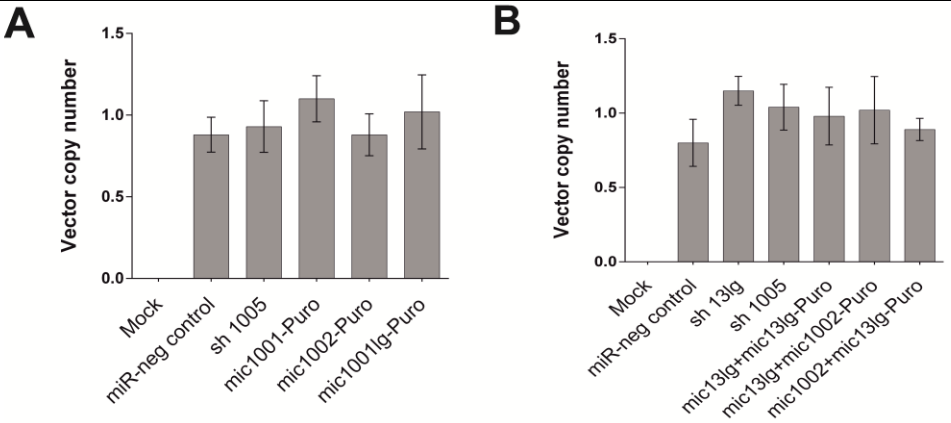


**Supplementary Figure S1.** Average transgene copy number per cell in transduced HT1080 CCR5-EGFP cells were determined by qPCR using previously described primers and probes [17]. The β-globin gene was used for normalization of the quantitative results. A - copy number for experiments described in Fig.1c. B - copy number for experiments described in Fig.2a.

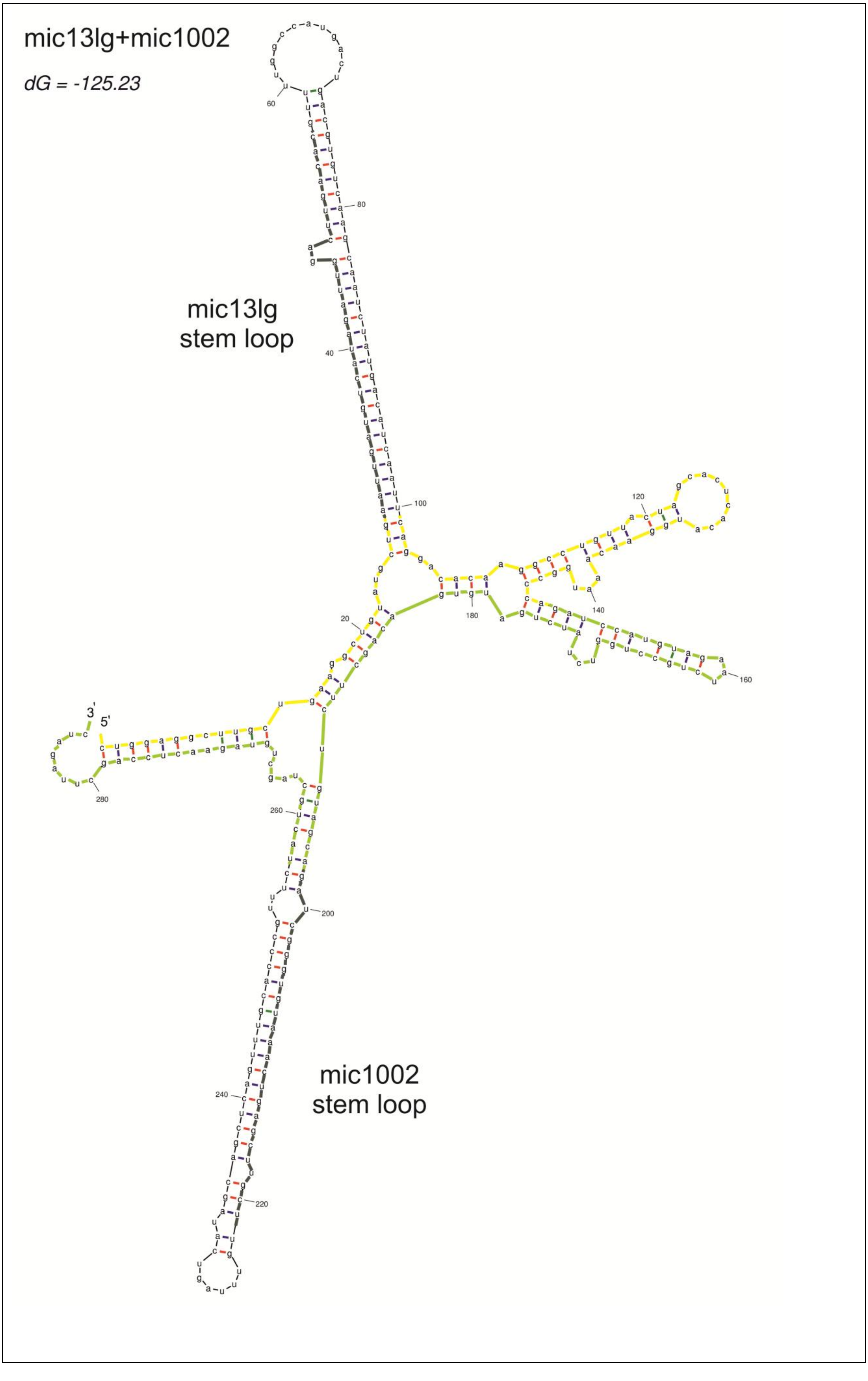
mic13lg+mic1002
dG = -125.23
mic13lg stem loop
mic1002 stem loop
3'
5'

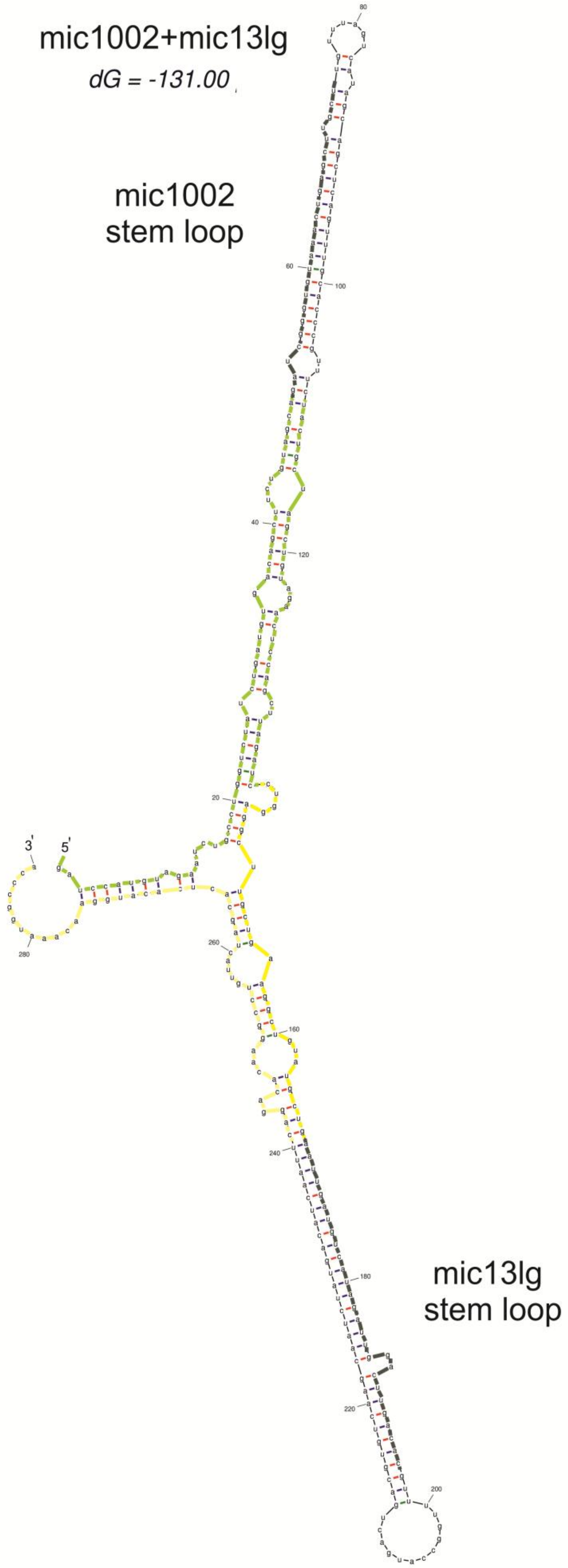
mic1002+mic13lg
dG = -131.00
mic1002
stem loop
mic13lg
stem loop
3'
5'
20
40
60
80
100
120
160
180
200
220
240
260
280

**Supplementary Figure S2.** Secondary structures of mic13lg+mic1002 and mic1002+mic13lg polycistrons obtained by folding of RNA sequences using the M-fold web server (http://unafold.rna.albany.edu/?q=mfold). mic13lg and mic1002 flanking sequences are depicted by yellow and green lines, respectively. Antisense chain in both amiRNA is shown as a black bold line. Stem-loop strutures of mic13lg and mic1002 including their antisense guide chains are identical for both polycistrons. Shuffling of two amiRNA sequences in polycistron caused structural changes in the amiRNA flanking region. These structural differences may affect the processing of amiRNA precursor, and therefore, the level of mature amiRNA in the cells.

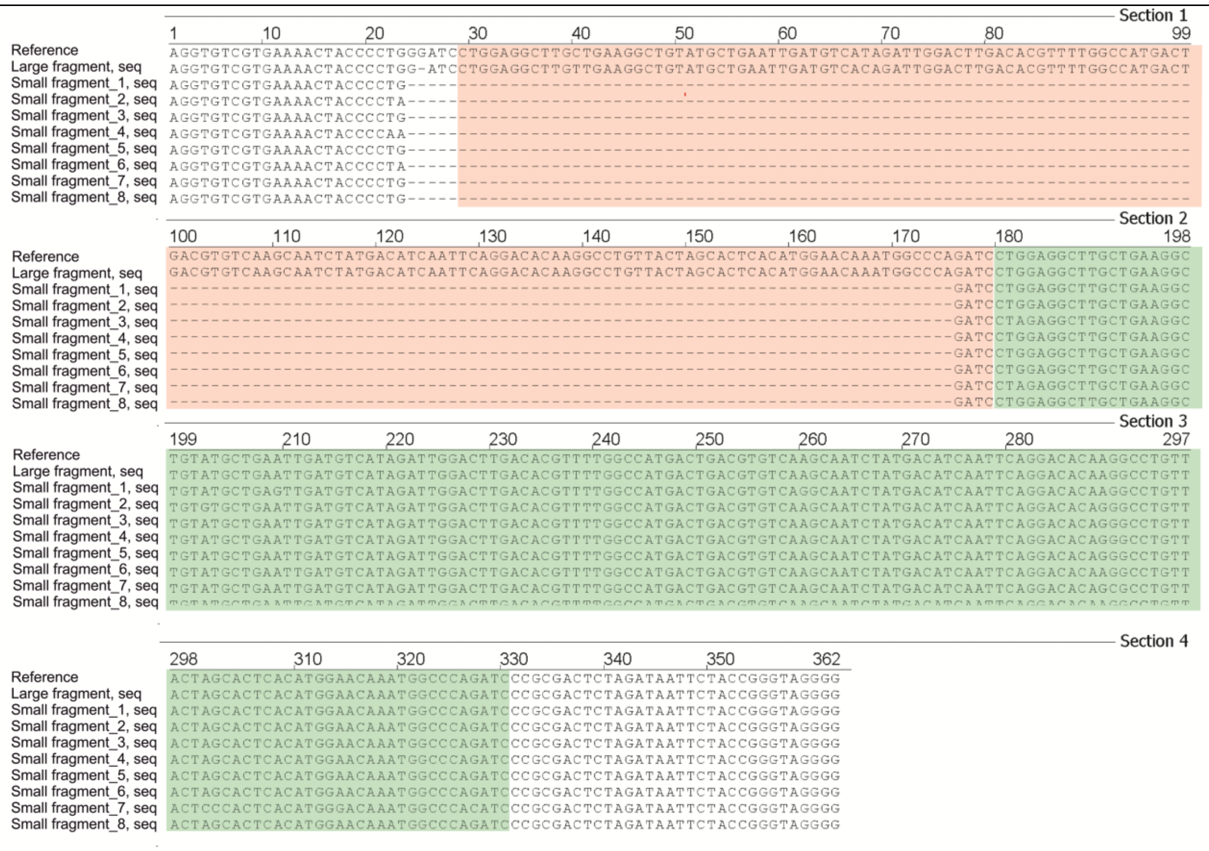


**Supplementary Figure S3.** Sequence analysis of PCR fragments (depicted in Figure 3F) which were generated by amplification of genome DNA of amiRNAs-transduced $CD4^+$ lymphocytes. All products of the PCR reaction were cloned into T-vector and single clones were analyzed by Sanger sequencing. Sequencing confirmed the presence of tandem amiRNA in long fragments and the presence of only one copy of single amiRNA in short fragments. First mic13lg amiRNA repeat is highlighted in orange, second repeat depicted in green color.

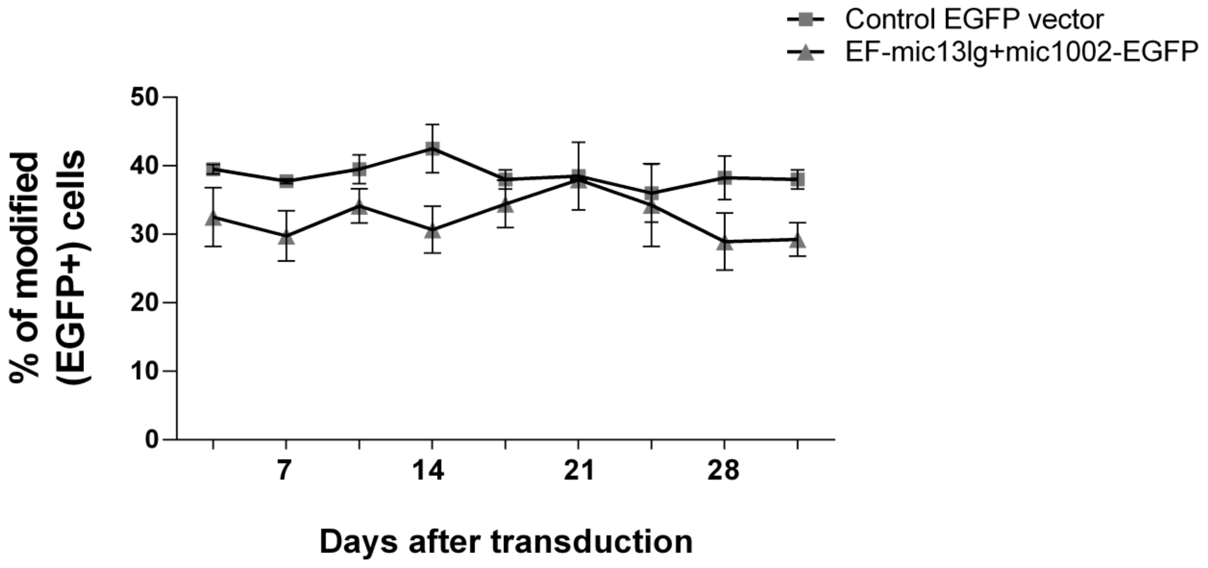


**Supplementary Figure S4.** Percentage of EF-mic13lg+mic1002-EGFP vector-modified $CD4^+$ cells ($EGFP^+$) remains stable during one month of *in vitro* culture. The fractions (%) of GFP-positive cells was measured over time in EF-mic13lg+mic1002-EGFP vector transduced cells and in the cells transduced with control EGFP vector.
